# Depletion of the cisplatin targeted HMGB-box factor UBF selectively induces p53-independent apoptotic death in transformed cells

**DOI:** 10.18632/oncotarget.4823

**Published:** 2015-08-07

**Authors:** Nourdine Hamdane, Chelsea Herdman, Jean-Clement Mars, Victor Stefanovsky, Michel G. Tremblay, Tom Moss

**Affiliations:** ^1^ Laboratory of Growth and Development, St-Patrick Research Group in Basic Oncology, Cancer Division of the Quebec University Hospital Research Centre, Québec, QC, Canada; ^2^ Department of Molecular Biology, Medical Biochemistry and Pathology, Faculty of Medicine, Laval University, Québec, QC, Canada; ^3^ Present address: Inserm, U1110, Institute of Viral and Liver Diseases, Strasbourg, France

**Keywords:** Upstream binding factor (UBF/UBTF), ribosome biogenesis, oncogenic stress, apoptosis, cisplatin

## Abstract

Cisplatin-DNA adducts act as strong decoys for the Upstream Binding Factor UBF (UBTF) and have been shown to inhibit transcription of the ribosomal RNA genes by RNA polymerase I. However, it is unclear if this plays a significant role in the chemotherapeutic activity of cis- or carboplatin. We find that cisplatin in fact induces a very rapid displacement of UBF from the ribosomal RNA genes and strong inhibition of ribosomal RNA synthesis, consistent with this being an important factor in its cytotoxicity. Using conditional gene deletion, we recently showed that UBF is an essential factor for transcription of the ribosomal RNA genes and for ribosome biogenesis. We now show that loss of UBF arrests cell proliferation and induces fully penetrant, rapid and synchronous apoptosis, as well as nuclear disruption and cell death, specifically in cells subjected to oncogenic stress. Apoptosis is not affected by homozygous deletion of the *p53* gene and occurs equally in cells transformed by SV40 T antigens, by *Myc* or by a combination of *Ras* & *Myc* oncogenes. The data strongly argue that inhibition of UBF function is a major factor in the cytotoxicity of cisplatin. Hence, drug targeting of UBF may be a preferable approach to the use of the highly toxic platins in cancer therapy.

## INTRODUCTION

The commonly used chemotherapeutic drugs cisplatin and carboplatin are generally considered to exert their cytotoxicity by inducing DNA damage. These drugs interact with DNA to form intra- and inter-strand crosslinks, which must be repaired for the cell to proliferate [1]. Hence, cells that grow more rapidly or are limited in their capacity to repair DNA should disproportionately suffer cell death, which often occurs by apoptosis. Consequently, growth factor driven tumour growth and deficits in the ability to rapidly repair DNA both enhance the ability of cisplatin to induce cell death [1–5]. DNA-platin adducts are also aberrantly bound by a range of nuclear proteins, and this in general enhances cell death by delaying their repair [6, 7]. Important among these nuclear proteins are members of the High Mobility A and B families (HMGA and HMGB), which display elevated affinities for the bent DNA structure of the platin adducts via their HMGA-box and HMGB-box DNA binding domains [8–10]. Upstream Binding Factor (UBF) is an abundant multi-HMGB-box transcription factor that defines the active state of ribosomal RNA (rRNA) gene chromatin by replacing the core histones and is essential for transcription of these genes [11–13]. It has long been known that UBF has a particularly high affinity for cisplatin-DNA adducts, which may act as molecular decoys to attract this factor away from the rRNA genes and in so doing suppress their transcription [14–19]. Since transcription of the rRNA genes is the central event in the assembly of ribosomes, the protein factories of the cell, their activity is essential for cell growth and proliferation. The ability of cisplatin adducts to act as decoys for UBF binding could, therefore, enhance the drugs cytotoxicity either by inhibiting DNA repair, by inhibiting ribosome assembly, or both.

The rRNA genes are transcribed by RNA polymerase I (RPI/PolI), which is dedicated to this task. UBF is an HMGB-box DNA binding protein and one of the two essential RPI basal transcription factors [11, 20–23]. UBF is generally thought to mediate binding of the pre-initiation factor SL1/TIF1B and pre-initiation complex (PIC) assembly at the rRNA gene promoter. But UBF also forms a nucleosome-like structure that replaces histone chromatin throughout the transcribed regions of the rRNA genes and is able to regulate RPI transcription elongation in response to growth factor signalling [11, 24–28].

Ribosomal biogenesis is the process by which ribosomal RNA (rRNA) is transcribed, processed and assembled with the ribosomal proteins to create ribosomes [21, 29]. This energy consuming process is accomplished in the nucleolus and requires the action of the three RNA polymerases along with more than 200 different proteins and several hundred snoRNP complexes. Regulation of ribosome synthesis constitutes a major determinant of the increased protein synthesis needed for cell proliferation and, as such, its up-regulation occurs in many cancers [30, 31]. An increased nucleolar volume reflects this increased ribosome synthesis, and is therefore a biomarker of cancer that was recognized already 80 years ago [32–34]. In fact, rRNA transcription is a common and probably an essential target of many oncogenes (Myc [35, 36], SV40-T antigen [37, 38] and the Ras and mTOR signalling pathways [39–43]), and tumour suppressors (p53 [44], ARF [45–47], Rb [48, 49] and PTEN [50]).

Ribosomal biogenesis is such a central process in cell growth that it is also under the direct surveillance of the p53 pathway [51]. Defects in rRNA gene transcription [52], rRNA processing [53] or ribosome assembly [54] all cause p53 stabilization and arrest of cell proliferation. These findings have led to the investigation of small molecule inhibitors of ribosomal transcription as potential chemotherapeutic agents. Inhibition of the RPI pre-initiation factor SL1/TIF1B [55] or induced proteasome degradation of the RPI large subunit [56] both lead to arrest of rRNA synthesis and mediate cell death dependent on p53 function. However, the key to successful cancer therapy remains the selective targeting of cancer cells, and since p53 is often inactivated in human cancers, therapies that depend on functional p53 have limited application. Our data now suggest that inhibition the RPI basal transcription factor UBF (Upstream Binding Factor) represents a particularly valuable p53-independent target for cancer therapy.

Here we show that displacement of UBF and ablation of rRNA synthesis are very early effects of cisplatin treatment, and that in the absence of cisplatin, elimination of UBF protein is sufficient to induce fully penetrant apoptotic cell death. Using cell cultures conditional for UBF expression, we find that complete loss of ribosome biogenesis induces synchronous and fully penetrant, p53-independent cell death by apoptosis specifically in cells transformed by known oncogenes. The data argue that a major factor in the cytotoxicity of cisplatin and similar drugs is their ability to inhibit the function of UBF. This suggests that UBF itself represents a preferred target for anticancer drug development.

## RESULTS

Previous data has clearly indicated that cisplatin treatment of human cells leads to a partial or full displacement of human UBF and inhibition of rRNA synthesis [14, 15, 17, 18]. However, to what extent this plays a role in the selective cytotoxicity of cisplatin is not known. When the Mouse Embryonic Fibroblast (MEF) derived cell line NIH3T3 was treated for 4 h with 30 μM cisplatin, a concentration calculated to be equivalent to the dose commonly used in therapy (e.g see [57, 58]), a large proportion of endogenous UBF was displaced from nucleoli and scattered throughout the nucleus at a large number of foci (Figure S1). These foci were devoid of the other nucleolar proteins fibrillarin and RPI (data not shown), which remained together in dense nuclear bodies somewhat similar to the nucleolar precursor bodies forming on conditional deletion of the *Ubf* gene [11].

### Cisplatin displaces UBF from the mouse rRNA genes and arrests their transcription

**Figure 1:**
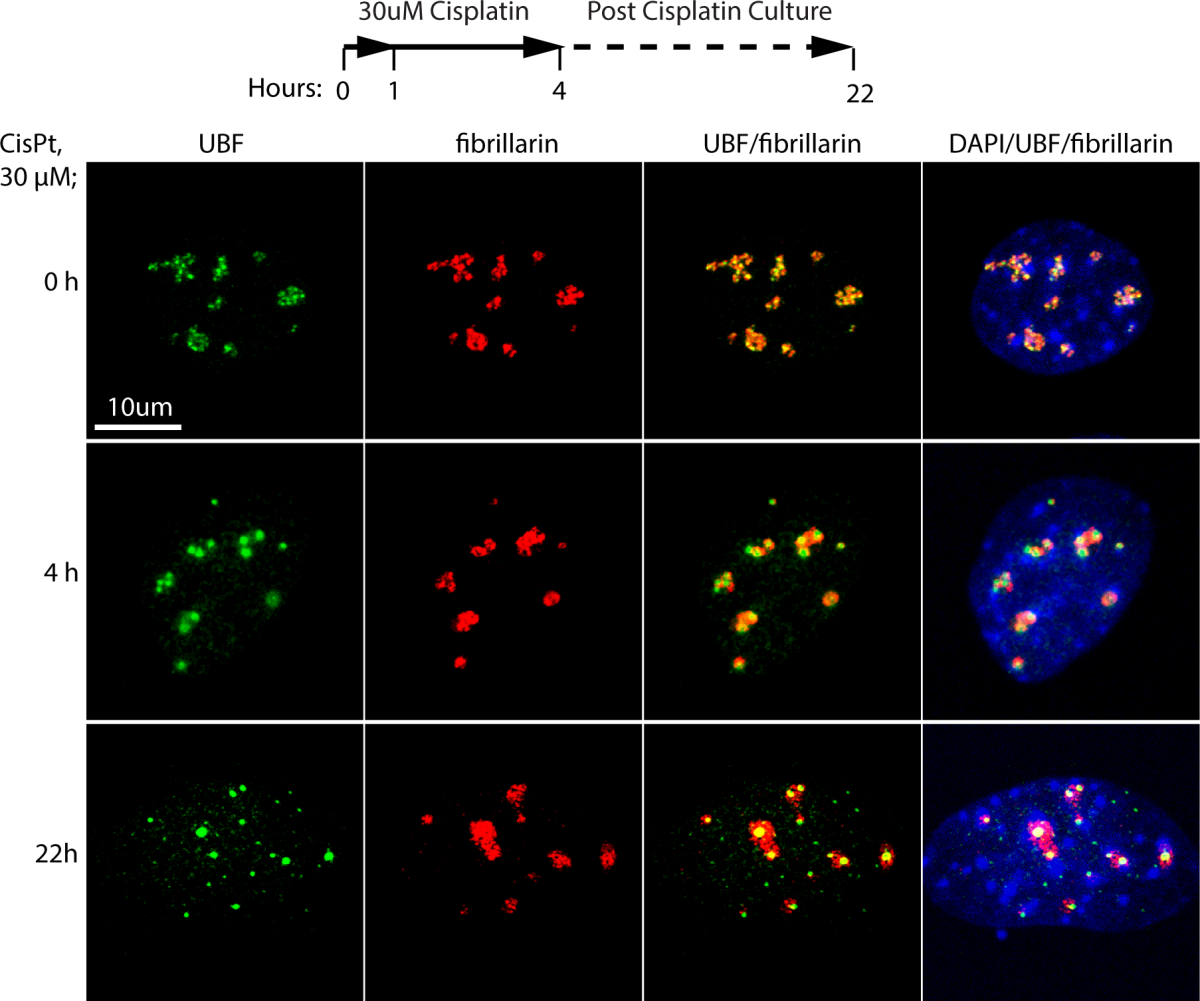
Cisplatin treatment of *Ubf^wt/wt^*/*Er-cre*^+/+^/*SvT* iMEFs induces displacement of UBF from the nucleolus iMEFs were treated with 30 μM cisplatin for 4 h in full medium or left untreated (0), then either fixed immediately or cultured in fresh medium lacking cisplatin overnight (22 h) as indicated in the timeline before fixing. The fixed samples were then subjected to indirect immunofluorescence analysis of UBF (green), fibrillarin (red) and DNA stained with DAPI (blue).

**Figure 2:**
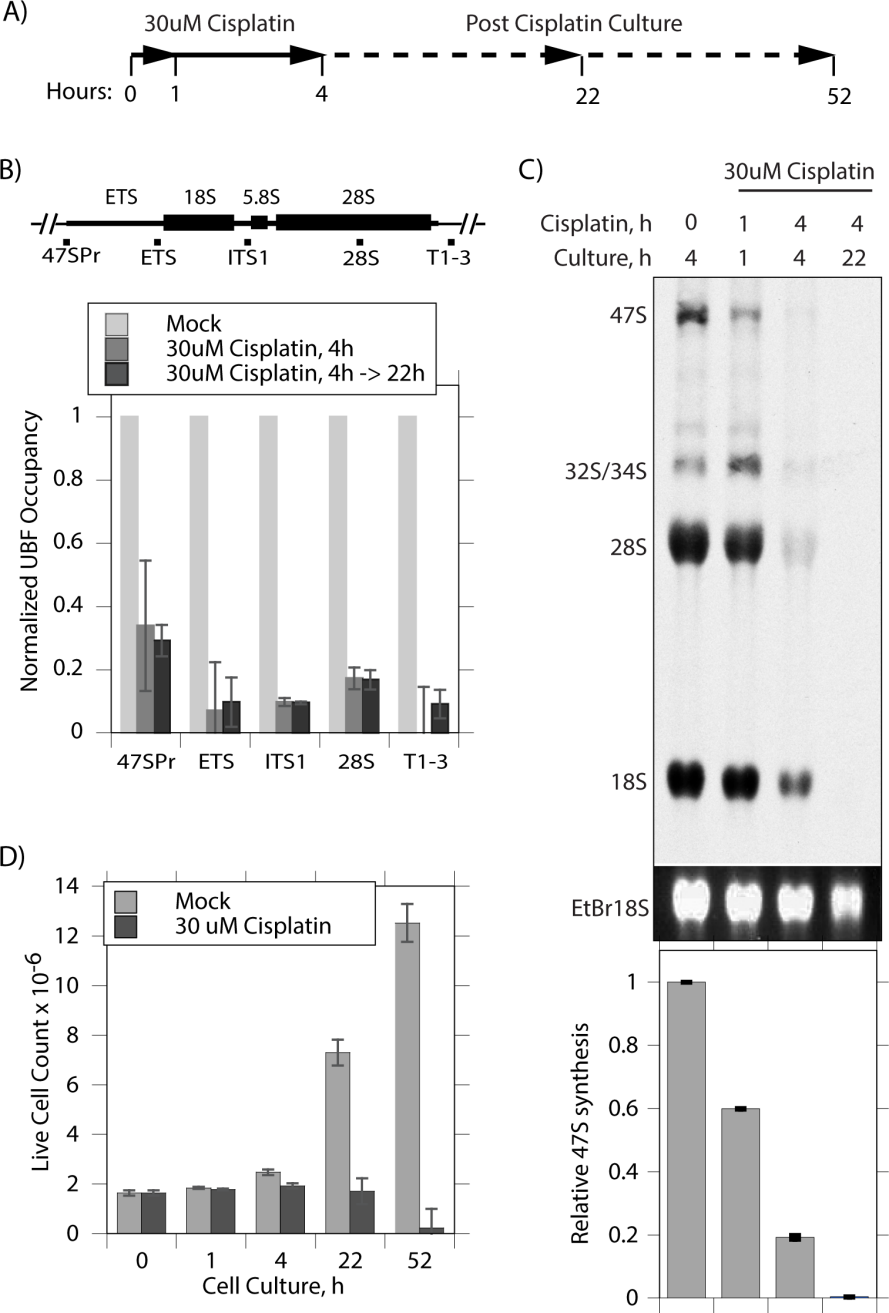
Cisplatin coordinately displaces UBF from the rRNA genes and arrests their transcription **A.** Timeline of cisplatin treatment and culture of *Ubf^wt/wt^*/*Er-cre*^+/+^/*SvT* iMEFs. **B.** ChIP analyses of UBF occupancy across the rRNA gene 47S transcribed region. The positions of amplicons is indicated above the histogram showing the UBF occupancy normalized to that in the mock treated cells. **C.** Synthesis rate of rRNA determined by [^3^H]-uridine metabolic labelling of mock treated cells and at the indicated times post cisplatin treatment. The upper panel displays a fluorogram of [^3^H]-rRNA, the central panel the corresponding EtBr stained total 18S rRNA, and the lower panel quantitation of [^3^H] incorporation into 47S rRNA performed in triplicate. **D.** Live cell counts at indicated times following cisplatin treatment performed in triplicate.

To better understand the effect of cisplatin, we repeated and extended these studies using the independently isolated, iMEF cell line (*Ubf^wt/wt^*/*Er-cre*^+/+^/*SvT*) previously characterized by Hamdane et al. [11]. Already after 4 h exposure of these cells to 30 uM cisplatin, UBF was seen to coalesce from its normal specular distribution within nucleoli into more intense foci, while fibrillarin showed some degree of coalescence but was less affected (Figure 1). When these cells were cultured for a further ~18 h in the absence of cisplatin, the UBF foci became more intense and UBF, but not fibrillarin, formed foci throughout the nucleus. The timing of the changes in UBF delocalization corresponded closely with changes in the interaction of UBF with the rRNA genes and with the transcription of these genes (Figure 2). After 4 h of cisplatin treatment a mean reduction in UBF binding of 80% was observed across the 47S precursor rRNA coding region, and this corresponded with an 80% reduction in rRNA synthesis (Figure 2B and 2C). (Due to its 5′ position in the 47S precursor, 18S rRNA synthesis was slightly less affected at 4 h than the 28S rRNA, but nevertheless was reduced by over 60% after 4 h cisplatin exposure, data not shown). 22 h after cisplatin exposure rRNA synthesis was no longer detectable. The effects of cisplatin on the activity of the rRNA genes also corresponded to an arrest of cell proliferation, no increase in the viable cells count being detected after the 4 h cisplatin treatment, and to a subsequent loss of viability (Figure 2D). These data suggest that the timeline of cisplatin cytotoxicity is consistent with its effects being mediated at least in part by disruption of UBF function, and the arrest of rRNA gene transcription and, hence, of ribosome biogenesis. Since cisplatin is a key chemotherapeutic agent that acts by inducing apoptotic cell death somewhat selectively in transformed cells (e.g. [3]), we sought to determine whether or not this activity could also be explained by the inhibition of UBF function.

### UBF loss disrupts nucleolar functions in both primary and transformed MEFs

We previously generated mice conditional for the *Ubf* gene and demonstrated that loss of this gene arrested mouse development at the morula stage [11]. SV40Tt immortalized Mouse Embryonic Fibroblasts or iMEFs (*Ubf^fl/fl^*/*Er-cre*^+/+^/*SvT*) generated from these mice allowed us to show that UBF was essential for transcription of the rRNA genes and for the existence of a functional nucleolus [11]. Not surprisingly, despite their limited proliferation potential, primary MEFs derived from these mice also require UBF for rRNA synthesis and for the maintenance of nucleoli (Figure S2). Thus, UBF loss in primary MEFs recapitulated the effects observed in the transformed iMEFs.

### Transformed iMEFs, but not primary MEFs, undergo synchronous apoptosis following *Ubf* inactivation

Despite the apparently identical responses of the primary MEFs and the iMEFs to UBF loss, it became obvious from observing these cultures that the two cell types behaved very differently macroscopically. Inactivation of rRNA gene transcription in the *Ubf^fl/fl^*/*Er-cre*^+/+^/*SvT* iMEFs induced changes in cell morphology soon after complete UBF depletion and the shutdown of rRNA synthesis. iMEFs became highly elongated and this presaged cell death as determined by plasma membrane failure (trypan blue), mitochondrial membrane depolarization (MitoTracker) and loss of clonal viability (Figure S3A to S3D). Control *Ubf^wt/wt^*/*Er-cre*^+/+^/*SvT* iMEFs suffered none of these effects, clearly demonstrating that cell death was exclusively the result of inactivation of the *Ubf* gene. Interestingly, we detected no selective reduction of total cellular RNA in the *Ubf^fl/fl^*/*Er-cre*^+/+^/*SvT* iMEFs relative to their wild type counterparts during UBF depletion that might suggest a role of ribosome depletion in the selective induction of apoptosis (data not shown). In contrast to the behavior of the *Ubf^fl/fl^*/*Er-cre*^+/+^/*SvT* iMEFs, the primary *Ubf^fl/fl^*/*Er-cre*^+/+^ MEFs showed no evidence of major morphological changes and survived in culture for many days following complete UBF loss, maintaining plasma membrane integrity and mitochondrial function (Figure S3A to S3C).

**Figure 3:**
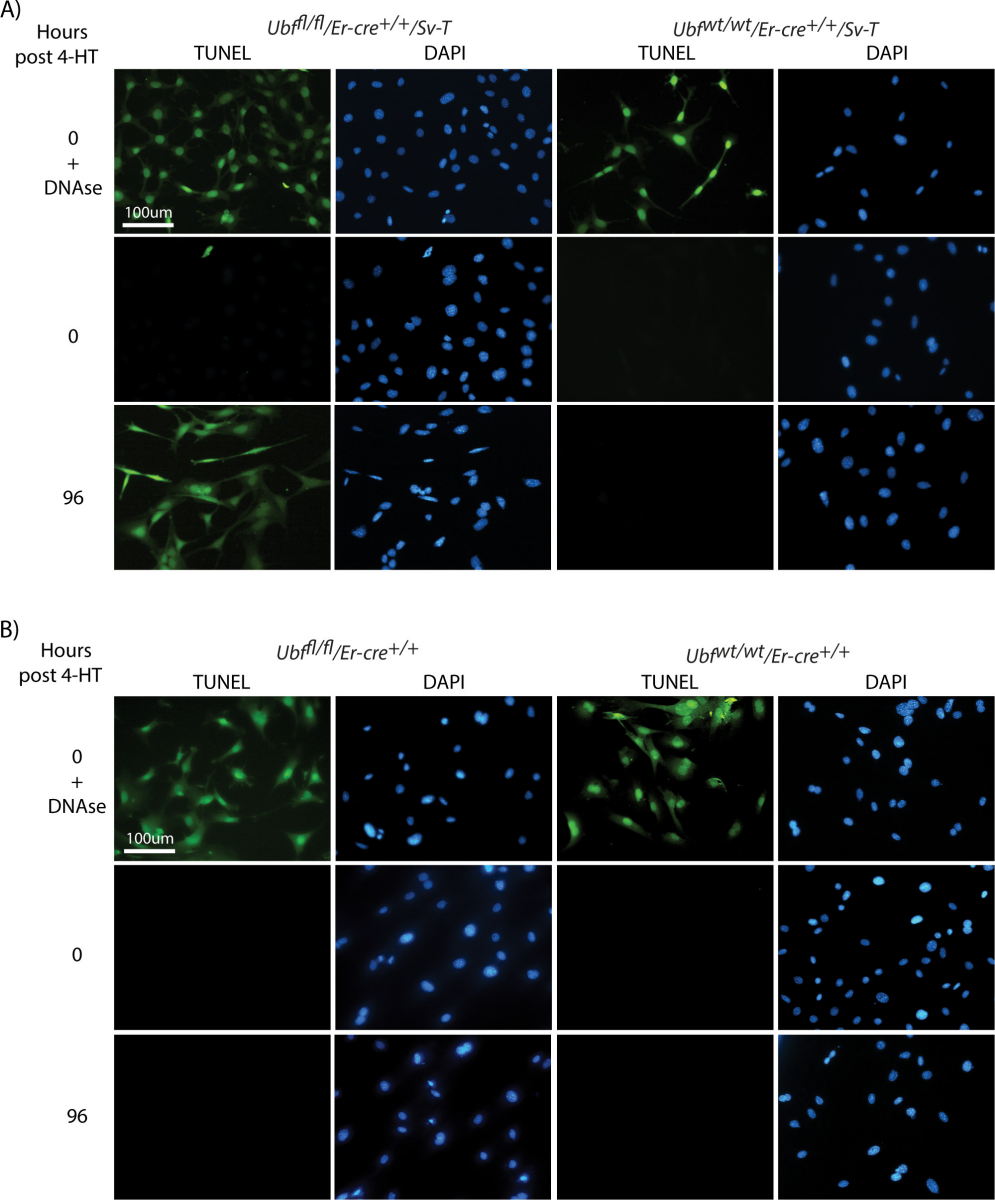
UBF loss induces synchronous apoptotic cell death selectively in oncogenically transformed iMEFs **A.** *Ubf^fl/fl^*/*Er-cre*^+/+^/*Sv-T* and *Ubf^wt/wt^*/*Er-cre*^+/+^/*Sv-T* iMEFs and **B.** *Ubf^fl/fl^*/*Er-cre*^+/+^ and *Ubf^wt/wt^/*Er-cre*^+/+^* primary MEFs were subjected to a TUNEL reaction immediately before, and at several time points after, treatment with 4-HT. In both cases, recombination and UBF protein levels were assayed in parallel and closely followed those shown in Figure S2B and S2C.

To better understand the different responses of the transformed iMEFs and primary MEFs to UBF loss, we analyzed them for typical markers of cell death. TUNEL (terminal deoxynucleotidyl transferase-mediated dUTP nick end-labeling) analysis detects the single strand DNA cleavage that is characteristic of the early stages of apoptotic cell death. *Ubf^fl/fl^*/*Er-cre*^+/+^/*SvT* iMEFs became TUNEL positive at 96 h pHT, just 24 h after complete shutdown of rRNA synthesis, while the control *Ubf^wt/wt^*/*Er-cre*^+/+^/*SvT* iMEFs remained TUNEL-negative throughout (Figure 3A). The TUNEL signal was fully penetrant and occurred synchronously, *Ubf^fl/fl^*/*Er-cre*^+/+^/*SvT* iMEFs being TUNEL-negative at 72 h pHT but all becoming TUNEL-positive at 96 h pHT. In contrast, the *Ubf^fl/fl^*/*Er-cre*^+/+^ primary MEFs remained TUNEL-negative at least until 144 h pHT, (Figure 3B and data not shown).

**Figure 4:**
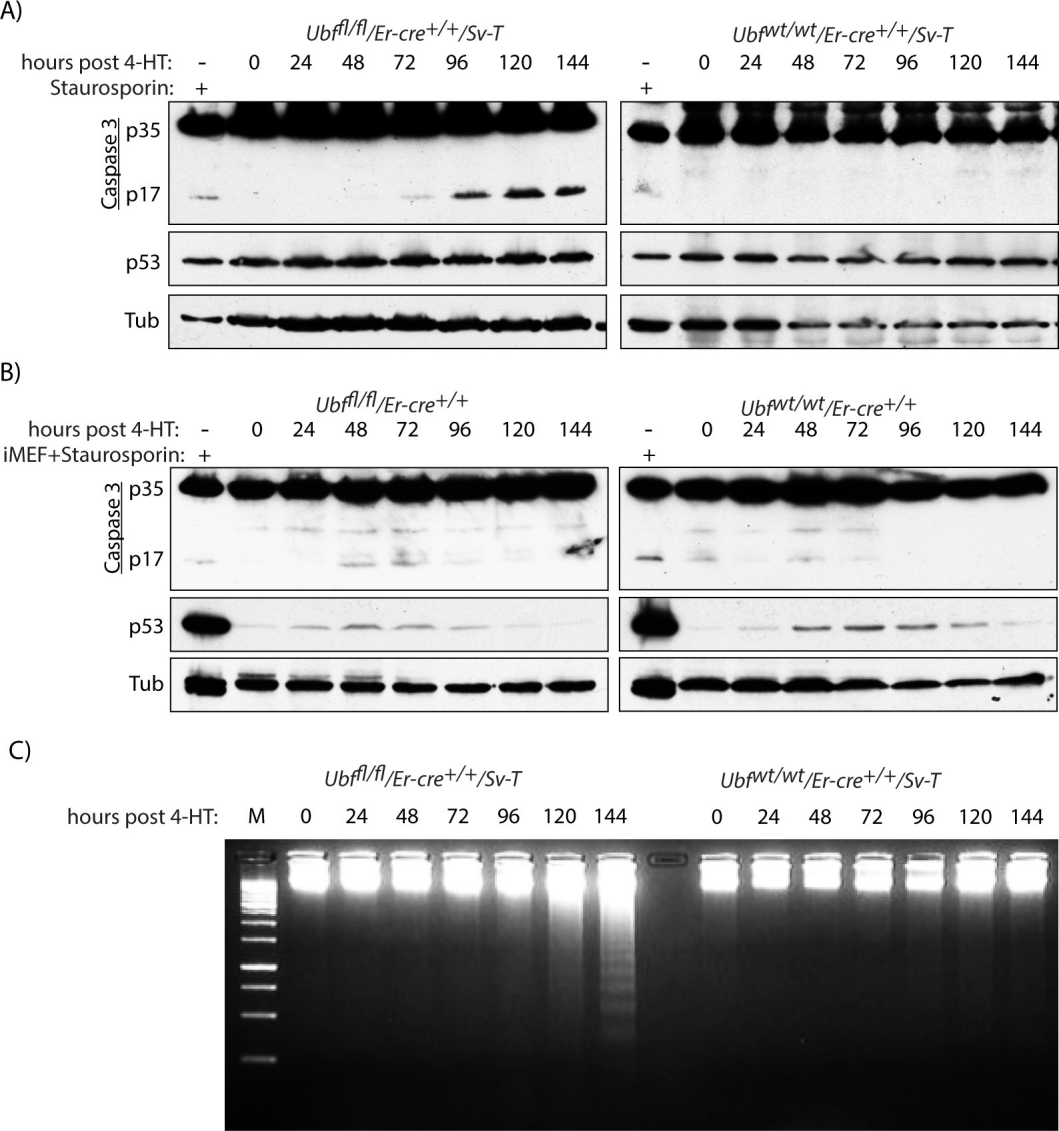
UBF loss induces selective Caspase 3 cleavage in transformed iMEFs cells **A.** *Ubf^fl/fl^*/*Er-cre*^+/+^/*Sv-T* and *Ubf^wt/wt^*/*Er-cre*^+/+^/*Sv-T* iMEFs and **B.** *Ubf^fl/fl^*/*Er-cre*^+/+^ and *Ubf^wt/wt^*/*Er-cre*^+/+^ MEFs were assayed for activation (proteolytic cleavage) of Caspase 3 immediately before and at time points after treatment with 4-HT. In B) “iMEF+Staurosporin” refers to the extract from iMEFs cells treated with 1 μM Staurosporin used in A, and allows a direct comparison of p17 and p53 levels in iMEFs with those in primary MEFs. **C.** Electrophoretic fractionation on 1.5% agarose of genomic DNA recovered from *Ubf^fl/fl^*/*Er-cre*^+/+^/*Sv-T* and *Ubf^wt/wt^*/*Er-cre*^+/+^/*Sv-T* iMEFs at different times post tamoxifen treatment (pHT). In A) to C), recombination and UBF protein levels were assayed in parallel with each analysis and closely followed those shown in Figure S2B and S2C.

Concomitant with the onset of TUNEL-positive apoptosis, the *Ubf^fl/fl^*/*Er-cre*^+/+^/*SvT* iMEFs were also found to activate Caspase 3 from 96 h pHT, as determined by the release of the 17kD peptide (p17) cleavage product (Figure 4A). In contrast, the control *Ubf^wt1wt^*/*Er-cre*^+/+^/*SvT* iMEFs displayed no significant cleavage of Caspase 3, consistent with the lack of a TUNEL signal. Further, Caspase 3 was not significantly activated in the primary MEFs (Figure 4B). Though a certain level of cleavage was detected in both *Ubf^fl/fl^* and *Ubf^wt/wt^* MEFs, this was much weaker than observed in the *Ubf^fl/fl^*/*Er-cre*^+/+^/*SvT* iMEFs as can be seen by comparison with Staurosporin-treated iMEFs.

Interestingly, unlike the deletion of UBF, deletion of the essential RPI initiation factor TIF1A/Rrn3 did not induce apoptosis in SV40Tt transformed MEFs. 4-HT treatment of *TIF1A^fl/fl^*/*Er-cre*^+/+^/*SvT*:MEFs resulted in complete depletion of TIF1A by 48 h pHT, as observed for UBF, but did not lead to activation of Caspase 3, nor to a TUNEL signal (Figure S4A and S4B). Thus, the induction of apoptosis in the SV40Tt transformed cells was not a general property of the arrest of rRNA gene transcription, suggesting it is specific to UBF depletion.

Given that the iMEFs were initially immortalized by the SV40 Tt oncogene (*Sv-T*), known to inactivate p53 [59, 60], it was not surprising to find the p53 levels in these cells were constitutively elevated and were not further induced by inactivation of the *Ubf* gene or by treatment with Staurosporin (Figure 4A). Thus, it was unclear whether or not p53 played a role in the apoptotic response in these cells. This question is directly addressed below using homozygous inactivation of the p53 gene. However, it should be noted that inactivation of the *Ubf* gene in the primary MEFs did not enhance the levels of p53 protein, which remained extremely low throughout (Figure 4B).

### Apoptosis is accompanied by the generation of a “nucleosomal ladder” of DNA cleavage

Apoptosis is often accompanied by inter-nucleosomal cleavage of genomic DNA to generate a “nucleosomal ladder” [61, 62], due to the result of the release of the nuclease EndoG from mitochondria [63, 64]. Beginning at or before 120 h pHT we observed this characteristic nucleosomal fragmentation of genomic DNA in the apoptotic *Ubf^fl/fl^*/*Er-cre*^+/+^/*SvT* but not in the control *Ubf^wt/wt^*/*Er-cre*^+/+^/*SvT* iMEFs (Figure 4C), nor in the corresponding primary MEFs (data not shown). Thus, three distinct markers; TUNEL signal, Caspase 3 cleavage and a nucleosomal ladder, indicated that on UBF loss MEFs underwent classic apoptotic cell death after oncogenic transformation with SV40-T, while UBF loss in untransformed MEFs induced none of these markers.

### UBF loss blocks proliferation and DNA replication, causing cell cycle arrest

**Figure 5:**
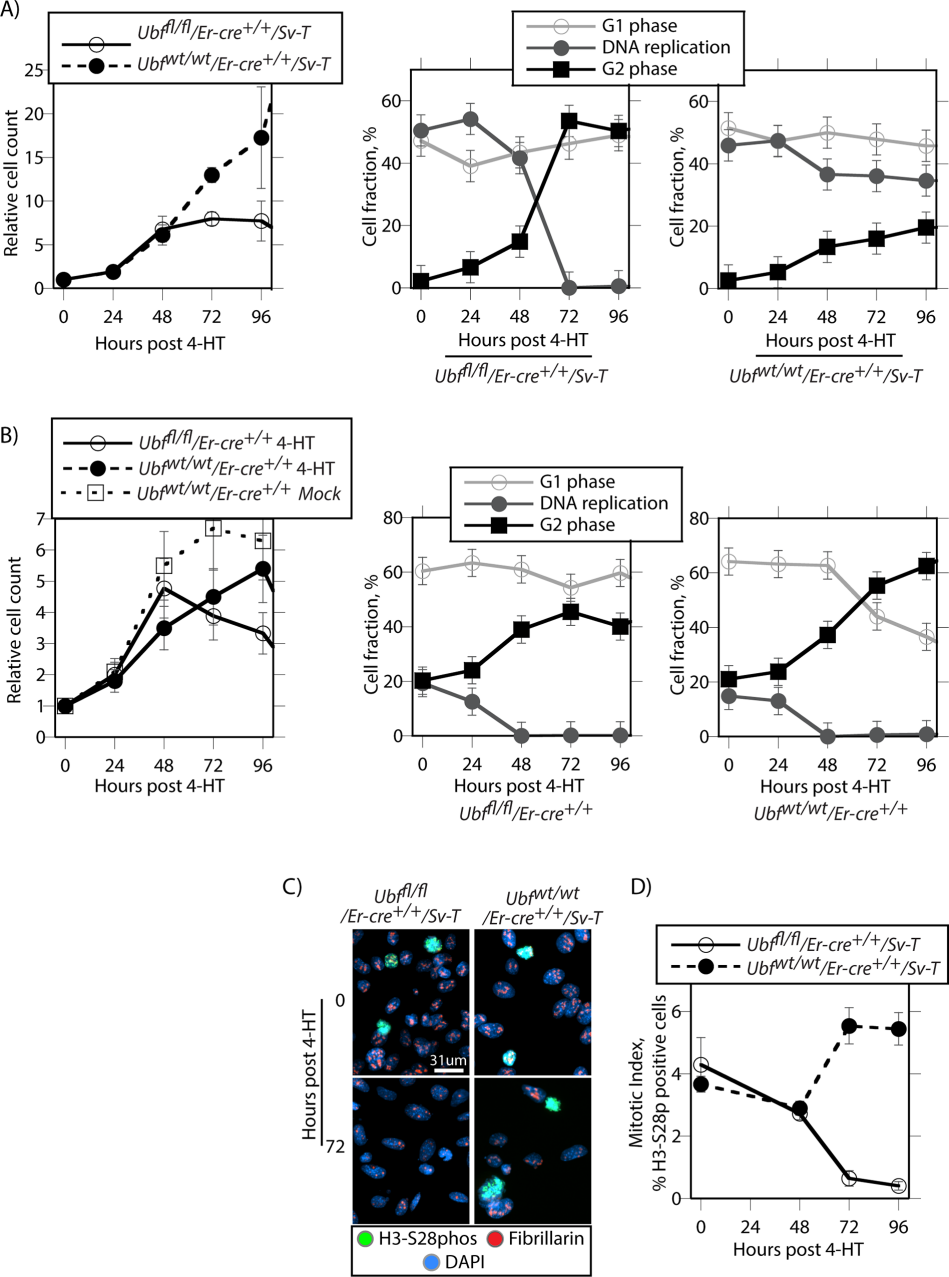
UBF loss arrests cell proliferation and leads to a cell cycle arrest **A.** *Ubf^fl/fl^*/*Er-cre*^+/+^/*Sv-T* and *Ubf^wt/wt^*/*Er-cre*^+/+^/*Sv-T* iMEFs and **B.** the corresponding primary MEFs were analyzed for proliferation and cell cycle distribution at the indicated times post 4-HT treatment. The left-most graphics give cell counts relative to day 0 and include those for *Ubf^wt/wt^*/*Er-cre*^+/+^ MEFs cultured in the absence of 4-HT (Mock), while to the right of these are shown the cell cycle distributions obtained from FACS analyses for active DNA replication (Click-iT^®^ EdU) and G1 and G2 DNA content (propidium iodide, PI). **C.** shows examples of mitotic staining, and **D.** a derived graphic of the mitotic index for the *Ubf^fl/fl^*/*Er-cre*^+/+^/*Sv-T* and *Ubf^wt/wt^*/*Er-cre*^+/+^/*Sv-T* iMEFs as determined by the fraction of H3-S28phospho positive cells. In A to D, *Ubf* recombination and UBF protein levels were assayed in parallel and closely followed those shown in Figure S2B and S2C.

To better understand the mechanisms leading to apoptosis in the transformed iMEFs, we determined the effects of *Ubf* inactivation on cell cycle progression and cell division. Before tamoxifen treatment, the *Ubf^fl/fl^*/*Er-cre*^+/+^/*SvT* iMEFs displayed a large (~50%) actively replicating S-phase population (Figure 5A). Their proliferation was near completely arrested by 48 pHT, corresponding with the elimination of UBF protein and with the near complete shutdown of rRNA synthesis (e.g. see Figure S2C to S2E and [11]). By 72 h pHT, iMEFs had also stopped active DNA replication and the G2 population abruptly increased at the expense of S-phase cells, while the fraction of G1/G0 cells remained constant (Figure 5A and S5A). Concomitantly, the mitotic index fell to zero as determined by the fraction of cells phosphorylated on serine 28 of histone H3 (H3-S28P) (Figure 5C and 5D and Figure S5B). Together these data suggested that many apparently G2 iMEFs were unable to complete their passage through mitosis. Parallel analysis of *Ubf^wt/wt^*/*Er-cre*^+/+^/*SvT* iMEFs post tamoxifen treatment revealed none of these effects, DNA replication and cell proliferation continuing essentially unabated (Figure 5A, 5C & 5D and S5B).

The situation was somewhat different in the primary *Ubf^fl/fl^*/*Er-cre*^+/+^ and control *Ubf^wtlwt^*/*Er-cre*^+/+^ MEFs (Figure 5B). These cells proliferated more slowly than iMEFs, and only a small fraction (~20%) was ever actively engaged in DNA synthesis. Further, regardless of UBF status these cells gradually arrested DNA replication between 24 h and 48 h pHT and displayed a corresponding increase in G2 cells, that is up to 24 h earlier than for the UBF-null iMEFs. Thus, the primary MEFs underwent a natural slowing or arrest of proliferation regardless of UBF status, while proliferation arrest in the iMEFs was a direct result of the loss of UBF protein. This suggested that the catastrophic cell death observed in the iMEF cultures was related to their inability to assume a quiescent state. In contrast, MEFs naturally arrested proliferation and became quiescent regardless of UBF status or 4-HT treatment (Figure 5B), and hence this may have protected them from cell death on inactivation of the *Ubf* gene. Essentially then, UBF loss specifically targeted the SV40-Tt transformed cells for apoptotic cell death, and what is more the effect was fully penetrant. This suggests that inhibition of UBF or of ribosome biogenesis might represent an ideal target for the development of cancer specific cytotoxic drugs.

### Apoptosis induced by UBF loss is p53 independent

**Figure 6:**
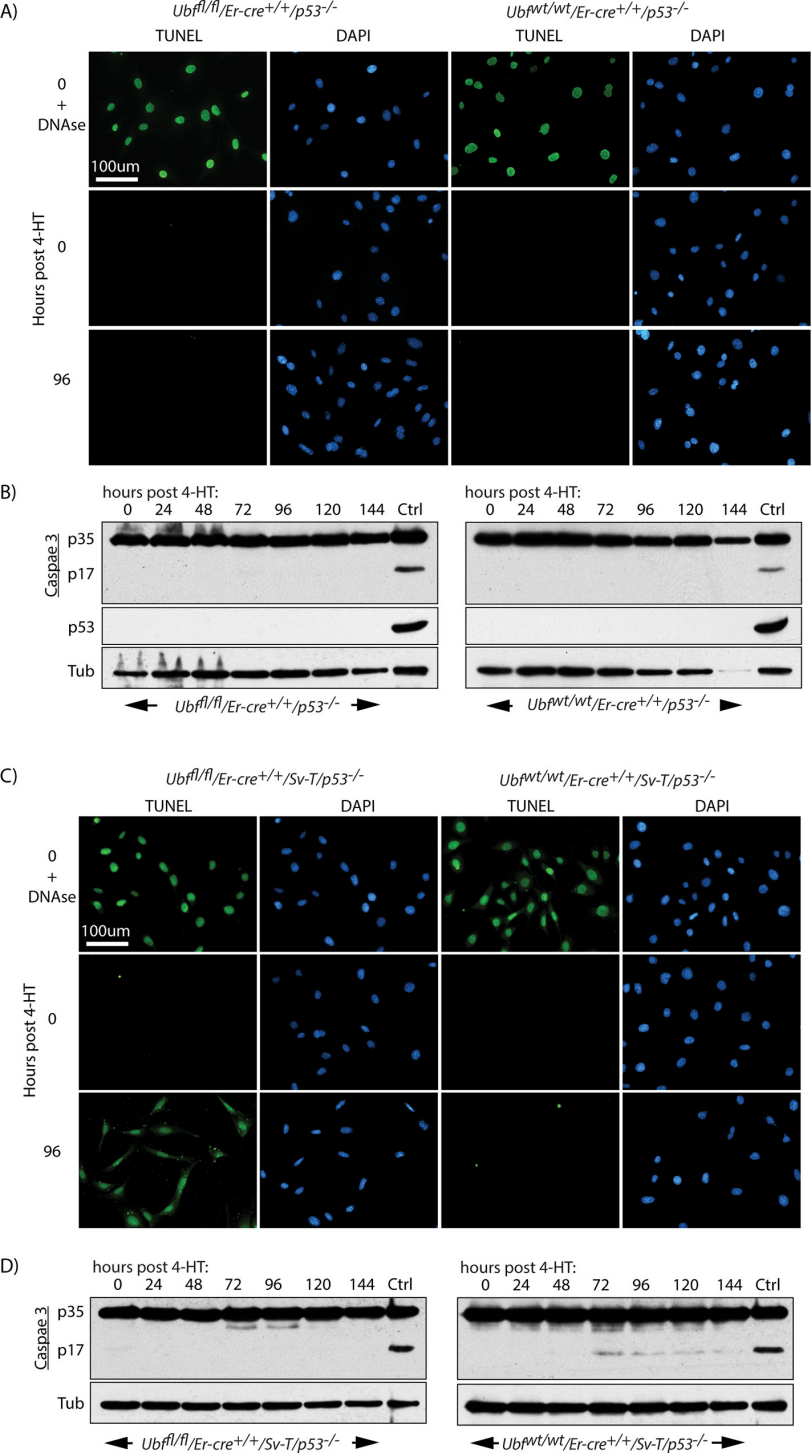
Apoptosis of oncogenically transformed cells after *Ubf* gene inactivation is p53 independent **A.** *Ubf^fl/fl^*/*Er-cre*^+/+^/*p53*^−/−^ and *Ubf^wt/wt^*/*Er-cre*^+/+^/*p53*^−/−^ MEFs and **C.** *Ubf^fl/fl^*/*Er-cre*^+/+^/*Sv-T/p53*^−/−^ and *Ubf^wt/wt^*/*Er-cre*^+/+^/*Sv-T/p53*^−/−^ iMEFs were subjected to a TUNEL reaction and **B.** and **D.** assayed for activation (proteolytic cleavage) of Caspase 3 immediately before and at several time points after treatment with 4-HT. P53-null iMEFs (*Ubf^fl/fl^*/*Er-cre*^+/+^/*Sv-T/p53*^−/−^) displayed the same TUNEL positive cell death, but Caspase 3 cleavage was not detected in these cells. In B) and D) “Ctrl” refers to an extract from iMEFs cells treated with 1 μM Staurosporin. Recombination of the *Ubf* gene and UBF protein levels were assayed in parallel and closely followed those shown in Figure S2B and S2C.

P53 is often required for the induction of apoptosis, hence its inactivation in many cancers represents a serious limitation to the efficacy of chemo- and radiation therapies [65–67]. The SV40 Tt oncogene is known to inactivate p53 [59, 60], suggesting that apoptosis induced by UBF loss did not depend on functional p53. To directly evaluate the role of p53, we generated p53-null MEFs either wild type or conditional for UBF (*Ubf^fl/fl^*/*Er-cre*^+/+^/*p53*^−/−^) (Figures 6 and S6A) and found that they were immortalized and hence could be passaged indefinitely. Despite this, they did not undergo apoptotic cell death on inactivation of the *Ubf* gene, and displayed neither a TUNEL signal nor Caspase 3 cleavage (Figure 6A and 6B). In contrast, after transformation with the SV40 Tt-antigens (SV40-T), the resulting p53-null (*Ubf^fl/fl^*/*Er-cre*^+/+^/*p53*^−/−^/*Sv-T*) iMEFs underwent synchronous and homogeneous TUNEL positive apoptosis two days after loss of UBF, exactly as observed for the p53 positive iMEFs (Figure 6C). Thus, even in the complete absence of p53 the loss of UBF was sufficient to induce apoptosis in the SV40-Tt transformed iMEFs. However, in this case no cleavage/activation of Caspase 3 was detected (Figure 6D).

### p53-independent apoptosis is a general response to UBF loss in oncogene stressed cells

**Figure 7:**
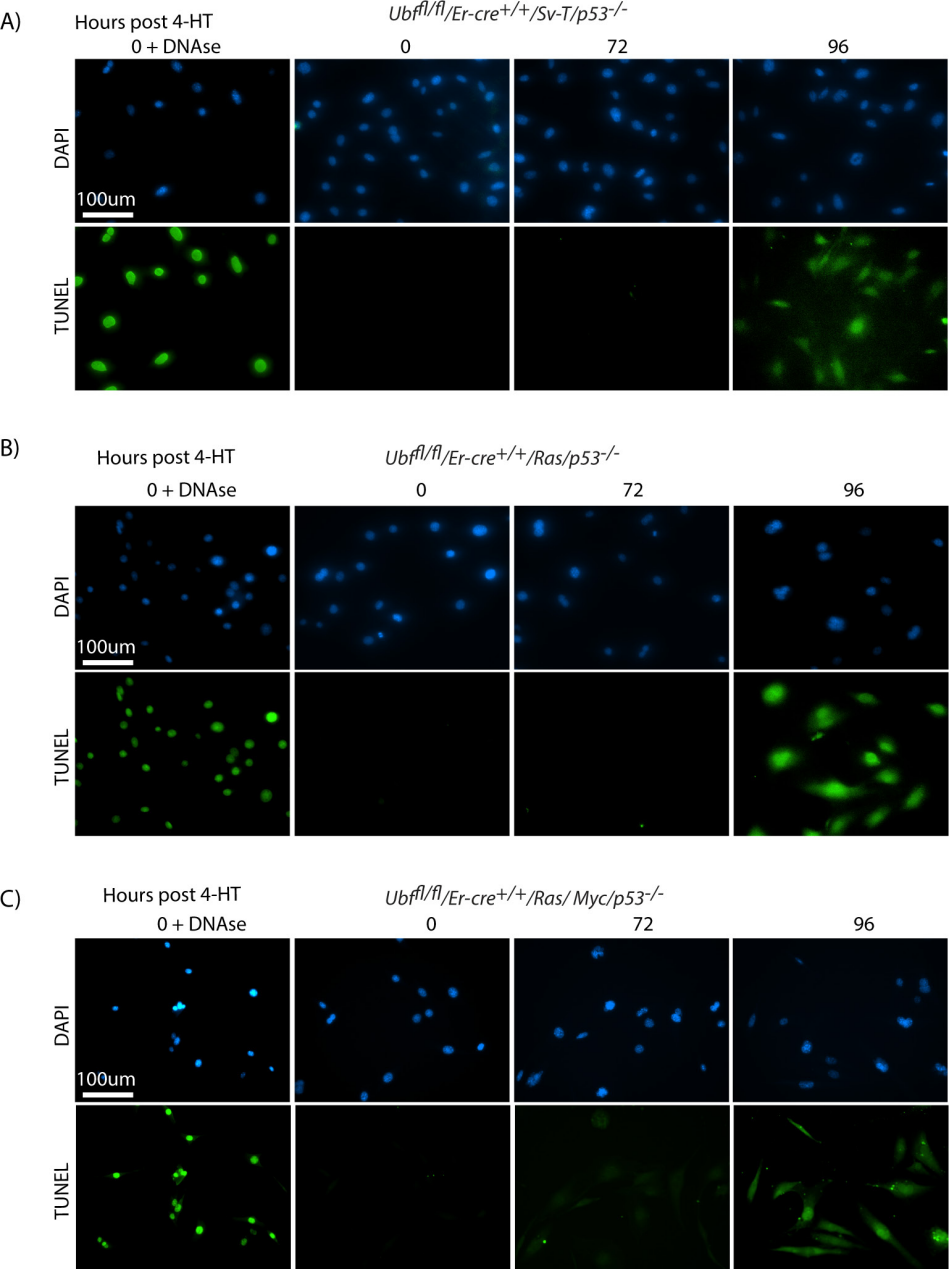
p53 independent apoptosis is a general response to UBF loss in an oncogenic stress context **A.** *Ubf^fl/fl^*/*Er-cre*^+/+^/*Sv-T/p53*^−/−^ and their counterpart **B.** *Ubf^fl/fl^*/*Er-cre*^+/+^/*Ras/p53*^−/−^ and **C.** *Ubf^fl/fl^*/*Er-cre*^+/+^/*Ras/Myc/p53*^−/−^ iMEFs cells were subjected to a TUNEL reaction immediately before and at several time points after treatment with 4-HT. All cells synchronously became TUNEL positive at 96 h post 4-HT, while neither effect was observed 24 h previously. Recombination of the *Ubf* gene and UBF protein levels were assayed in parallel and closely followed those shown in Figure S2B and S2C.

It was striking that UBF loss induced fully penetrant apoptosis in SV40-Tt transformed MEFs even in the complete absence p53. To determine if this effect was specific to the SV40-Tt oncogene or occurred under other oncogenic stresses, we investigated UBF-loss in MEFs transformed by the Ras and Myc oncogenes, commonly correlated with human cancers [68]. *Ubf^fl/fl^*/*Er-cre*^+/+^/*p53*^−/−^ MEFs were transformed by introduction of the Ras oncogene or a combination of the Ras and Myc oncogenes and the effects of inactivation of the *Ubf* gene were followed. In each case UBF was essentially eliminated by 48 h pHT (Figure S6B) and we observed a synchronous and homogeneous onset of TUNEL-positive apoptosis 48 h later, exactly as for SV40-Tt transformation (compare Figure 7A with 7B and 7C). Colony forming assays also showed that in each case cell death approached 100% (Figure S6D). In the case of SV40-Tt and combined Ras/Myc transformation we also observed a “nucleosomal ladder” of apoptotic DNA cleavage starting at 96 h pHT, that is at or just after the appearance of the TUNEL signal (Figure S6C), though this cleavage was not detected in the cells transformed with Ras alone.

### Oncogenic stress may induce apoptosis by aberrantly driving cells into S-phase

**Figure 8:**
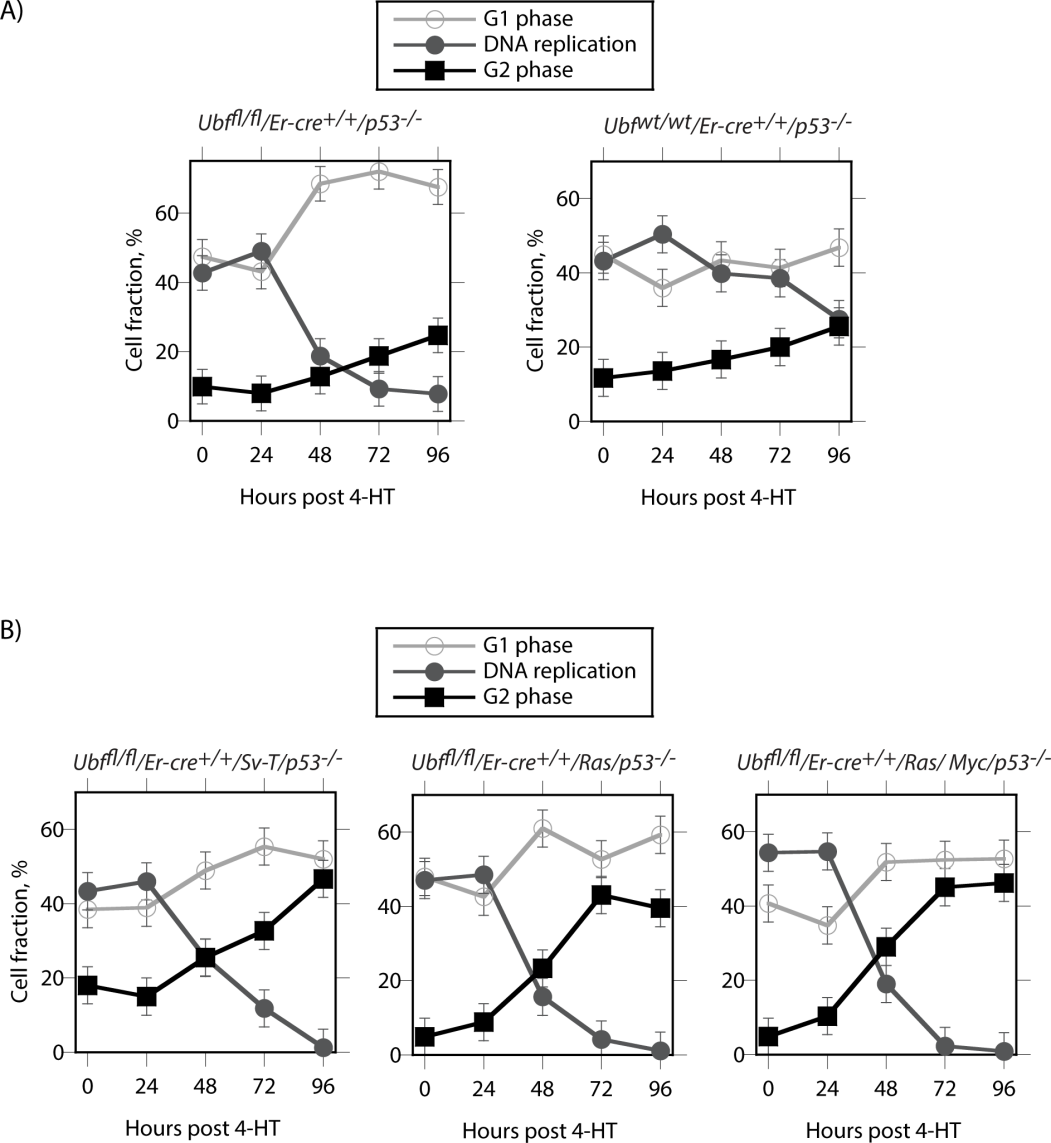
Cell cycle distribution of p53-null cells during UBF depletion **A.** Untransformed *Ubf^fl/fl^*/*Er-cre*^+/+^/*p53*^−/−^ and *Ubf^wt/wt^*/*Er-cre*^+/+^/*p53*^−/−^ MEFs. **B.** The same p53-null MEFs after transformation with SV40Tt, Ras or Ras plus Myc oncogenes. The graphics show the cell cycle distributions obtained from FACS analyses for active DNA replication (Click-iT^®^ EdU) and G1 and G2 DNA content (propidium iodide, PI).

When the untransformed p53-null cells (*Ubf^fl/fl^*/*Er-cre*^+/+^/*p53*^−/−^) were analyzed by FACS, we were surprised to find that, quite unlike the SV40-Tt transformed (*Ubf^fl/fl^*/*Er-cre*^+/+^/*p53*^+/+^/*Sv-T*) iMEFs (Figure 5A), UBF depletion caused a significant accumulation of cells in G1 at the expense of the actively replicating S-phase cells (Figure 8A). The G2 cell population displayed only a small increase and this anyhow closely resembled that observed for the control *Ubf^wt/wt^*/*Er-cre*^+/+^/*p53*^−/−^ cells. In contrast, the *Sv-T*, *Ras* and *Ras/Myc* transformed *Ubf^fl/fl^*/*Er-cre*^+/+^/*p53*^−/−^ cells displayed the same G2 phase accumulation as seen for the p53-positive iMEFs (compare Figure 8B with 5A). This suggested that transformation drives cells into and through S-phase regardless of their ability to generate a full complement of ribosomes. Such a situation would be likely to lead to gross replicative errors and hence could explain the highly penetrant apoptosis occurring in both the p53-positive and p53-null transformed MEFs, but not in the untransformed p53-null MEFs.

## DISCUSSION

Our data suggest that the ability of cisplatin to cause the displacement of UBF from the nucleolus is a key mechanism by which this drug induces selective cell death, since the simple loss of UBF induces a rapid and highly penetrant apoptosis in oncogenically stressed cells. We have shown that conditional deletion of the *Ubf* gene induces apoptosis specifically in cells transformed by viral and cellular oncogenes. Apoptosis following UBF loss was observed not only in cells expressing SV40Tt, but also in cells expressing the oncogenes Ras and Myc. What is more, in each case apoptosis was found to be fully penetrant, all cells without exception underwent apoptotic cell death. Strikingly, the onset of apoptosis occurred synchronously in all cells two days following complete loss of UBF. Significantly, the induction of TUNEL-positive cell death was completely independent of p53, since it occurred with the same timing and penetrance even after homozygous deletion of the *p53* gene. In contrast, before oncogenic transformation primary cell cultures survived complete loss of UBF for many days after the transformed cells entered apoptosis and never underwent apoptosis.

These data strongly suggest that the commonly used chemotherapeutic drug Cisplatin, and by analogy, Carboplatin exert their cytotoxicity in large part by hijacking UBF, displacing it from the nucleolus and inhibiting ribosome biogenesis. In fact, inhibition of ribosome biogenesis may be a more general property of the cytotoxic drugs used in chemotherapy than previously realized, including rapamycin analogs, 5-fluorouracyl and camptothecin [52, 69]. Azacytidine (Azacitidine, Vidaza) and deoxyazacytidine (Decitabine) are DNA methyltransferase inhibitors that have been shown to be active in treating myelodysplastic syndromes and acute myeloid leukemia (AML) [70–72]. The initial studies of azacytidine already showed that it strongly inhibits ribosome biogenesis, and almost certainly does so by preventing rRNA methylation [73, 74]. More recently, deoxyazacitidine was also shown to inhibit ribosome biogenesis by inhibiting rRNA processing, though the underlying mechanism of action is quite different and involves loss of rRNA gene silencing and aberrant RNA polymerase II transcription of these genes [13, 75]. Recent studies of small molecule inhibitors that target ribosome biogenesis have further shown this may be a very valid clinical approach to treating a range of cancers [55, 56, 76, 77]. However, while cell death was independent of p53 in the case of the GC-rich DNA interacting drug BMH-21 [56], it was found to be dependent on a functional p53 in the case of CX-5461, which is believed to target the pre-initiation complex factor SL1 [55]. Our data showing TIF1A/Rrn3-loss does not induce apoptosis even in the presence of p53 clearly excludes the explanation that the cytotoxicity of these drugs is simply a function of their ability to suppress rRNA synthesis. Why then inhibition of UBF can induce apoptotic cell death with such penetrance and in the complete absence of p53 is for the still a matter of conjecture. However, it is amost certainly related to the role of UBF in forming a specialized chromatin structure on the active rRNA genes [11]. Loss of this structure would yield the rRNA gene arrays highly susceptible to damage, and given the GC-richness of the rRNA genes the same could be argued for both cisplatin and BMH-21 drugs.

The Nucleolar Organizer Regions (NORs) each encompass around 40 rRNA gene units on the short arms of the five human acrocentric chromosomes [78]. These loci are particularly susceptible to DNA breakage and are subject to high levels of inter- and intra-chromosomal recombination [79–81]. Indeed, Robertsonian translocations have long been known to predominantly involve exchanges between the short arms of human acrocentric chromatids that often create fusions with chromatids of a metacentric chromosome [82]. Recent data strongly suggests that these and similar chromosome translocations result from disruption of the active chromatin structure of the rRNA genes, which in turn affects chromosome pairing causing aberrant resolution of mitotic chiasmata and fusion between non-homologous chromatids [83]. Loss of UBF clearly disrupts the chromatin structure of the rRNA genes, leaving them at least transiently as naked DNA, and would necessarily leave these genes highly susceptible to DNA damage and breakage. Since transformed iMEFs continue replication during UBF depletion, the disruption of rRNA gene chromatin would exacerbate the effects of DNA breakage, probably inhibit homologous repair processes and hence destabilize the genome. Indeed such destabilization has recently been observed as a result of siRNA knockdown of UBF [84].

## MATERIALS AND METHODS

### Isolation and cultures of MEFs and iMEFs

The generation of conditional *Ubf^fl/fl^Er-cre*^+/+^ and control mouse lines was previously described [11]. The p53-null allele was introduced by crossing to strain *129-Trp53^tm1Tyj^*/*J* (Jackson Laboratory Stock # 002080). Primary mouse embryonic fibroblasts (MEFs) from E14.5 *Ubf^fl/fl^*/*Er-cre*^+/+^ and isogenetic *Ubf^wt/wt^Er-cre*^+/+^ MEFs and corresponding *p53^−/−^* MEFs were prepared as previously described [11, 85]. Cells were cultured in Dulbecco's modified Eagle medium (DMEM)-high glucose (Life Technologies), supplemented with 10% fetal bovine serum (Wisent) and Antibiotic/Antimycotic (Wisent). Where indicated, Cisplatin (Sandoz) was added to the cell culture medium from a 100 mM solution in DMSO to give a final concentration of 30 μM and cells incubated for 4 hr at 37°C. The culture medium was then replaced with medium without cisplatin and cells incubated for a further 16 h at 37°C, before processing for immunofluorescence as described below. MEFs were immortalized by the introduction of the SV40 Tt antigens by transfection with the pBSV0.3T/t, a modification of the pBSV-early vector [86] kindly provided by E. W. Khandjian. The Ras and Ras/Myc transformed MEFs were generated by transfection or co-transfection with the plasmids pWZL-Ras-hygro and pBabe-c-myc-puro (kind gifts from Gerardo Ferbeyre) into *Ubf^fl/fl^*/*Er-cre*^+/+^/*p53*^−/−^ MEFs and subsequent hygromycin or double hygromycin/puromycin selection.

### Inactivation of *Ubf* or *Tif1a* in cell culture, and analysis of genotype, RNA and proteins

As previously described [11], cells were initially plated in 6 cm petri dishes (0.8 × 10^6^ cells each) and cultured for 18 hours in DMEM, high glucose, 10% fetal bovine serum. To activate ER-Cre, 4-hydroxytamoxifen (4-HT) was added to a final concentration of 50 nM, and after 4 hr incubation the medium replaced with fresh medium without 4-HT and cells harvested for analysis at various time points. In the case of *Tif1a*, cells were treated with 50 nM 4-HT, 0 h, then this treatment was repeated at 9 h, 24 h and 33 h later to ensure complete gene excision. Analyses of RNA, protein and genotype were systematically carried out on parallel cell cultures. Cells were genotyped by PCR before and after 4-HT treatment using the primers: A; 5′TGATCCCTCCCTTTCTGATG, B; 5′TGGGGATAGGCCTTAGAGAGA, C; 5′CACGGGAAAACAAGGTCACT, (Figure S2B). Metabolic labelling of RNA was carried out just before cell harvesting by addition of 10 μCi [^3^H]-uridine (PerkinElmer) to the culture medium and incubation for a further 3 h. RNA was extracted with Trizol (Life Technologies) according to the manufacturer's protocol and analyzed by gel electrophoresis, fluoroimaging (ENHance, PerkinElmer) and RNA species quantitated by scintillation counting as previously described [39, 40]. For total protein, cells were washed with cold PBS, scraped into PBS, centrifuged 30 s at 14 000 r.p.m., then resuspended in sodium dodecyl sulphate (SDS) loading buffer. After fractionation on 8%, 12% or 5–15% gradient SDS–polyacrylamide gel electrophoresis (SDS-PAGE [87]), cell extracts were analysed by standard Western blotting procedures.

### Chromatin immunoprecipitations (ChIP)

ChIP was performed as previously described [11, 88]. The amplicon coordinates relative to the 47S rRNA initiation site (BK000964) were as follows: 47SPr, 45133–40; ETS, 3078–3221; ITS1, 6258–6432; 28S, 10215–10411; T1–3, 13412–13607.

### Antibodies for western blot, immunofluorescence and ChIP

Rabbit antibodies against UBF, RPI large subunit (A194), TTF-1 and TIF1A were generated in the laboratory. All other antibodies were obtained commercially; Anti-Caspase-3, -p53 and -H3S-28phospho (Cell Signalling), anti-Tubulin (Sigma) and anti-Fibrillarin (Covance).

### Immunofluorescence

Cells were washed with PBS, fixed in 4% paraformaldehyde /PBS for 15 minutes and permeabilized with 0.5% Triton/PBS for 5 minutes. Incubation with primary antibody was performed for 1 h in PBS-5% BSA or 5% goat serum and cells were stained with AlexaFluor 488/568 conjugated anti-rabbit or -mouse IgG (Molecular Probes) and counterstained with DAPI. After mounting in 50% glycerol/50% 0.2 M Na-glycine, 0.3 M NaCl, 3D epifluorescent image stacks were generated on a Leica DMI6000B microscope equipped with a 63x or 100x objective and an Orca C4742–80-12AG camera (Hamamatsu). Image stacks were then deconvoluted and analyzed using Volocity software (Perkin-Elmer Improvision). Alternatively, image stacks were generated on a Leica SP5-II confocal microscope equipped with a 63x objective and running in standard scanning mode, and analyzed using Volocity software (Perkin-Elmer Improvision).

### FACs analysis and determination of Mitotic Index

Cells were stained for ongoing DNA synthesis using the Click-iT^®^ EdU Alexa Fluor^®^ 647 Flow Cytometry Assay Kit (Life Technologies) following the manufacturer's protocol and subsequently with propidium iodide (PI) immediately before analysis by the cytometry service of the CHU de Québec Research Centre using a FACSCanto II flow cytometer and FACSDiva 6.1.2 software (Becton Dickinson). Parallel cultures were stained with anti-H3S-28phospho antibody and DAPI and imaged by epifluorescence on the Leica DMI6000B microscope using 20 and 40x objectives. The Mitotic Index was calculated as the ratio of H3S-28phospho-positive to DAPI positive nuclei.

### Tunnel assays

Tunnel assays were performed with a Click-It Tunnel assay kit, Alexa 488 Imaging System, (Life Technologies). Cells were seeded in 35 mM petri dishes, fixed and processed according to the manufacturer's protocol and visualized by epifluorescence on the Leica DMI6000 B microscope using a 20x objective.

### Colony formation assays

The SV40-T, Ras and Ras/Myc transformed *Ubf^fl/fl^*/*Er-cre*^+/+^/*p53*^−/−^ and the isogenic wild-type MEF cells cultured in 100 mm petri dishes were treated with 50 nM 4-HT (Sigma) on day 0. The medium was changed after four hours to remove 4-HT, and on day 2 each culture was replated in duplicate at dilutions of 10 000, 50 000, 100 000, and 200 000 cells per 60 mm petri. On day 6 and day 12 petri dishes were fixed for 5 mins with 4% paraformaldehyde/PBS and stained with 0.05% crystal violet in distilled water (filtered) for 30 mins. Petri dishes were then washed 3 times with water and left inverted to dry before being photographed.

### MitoTracker assays

Cells were plated in Ibidi 35 mm thin bottom petri dishes for subsequent live cell microscopy and treated for 4 h with 50 nM 4-HT (Sigma) and further cultured as standard for 96 h to induce UBF loss. Cells were then treated with 25 nM MitoTracker DeepRedTM (Life Technologies) for 20 mins at 37°C in DMEM minus serum. Petri dishes were washed once with DMEM minus serum and then incubated in FluoroBrite DMEM (Life Technologies). Finally, live image stacks were generated on the Leica SP5-II confocal microscope and analyzed using Volocity software (Perkin-Elmer Improvision).

## SUPPLEMENTARY DATA FIGURES


