# Supplementary material for: Depletion of the cisplatin targeted HMGB-box factor UBF selectively induces p53-independent apoptotic death in transformed cells
Source: Oncotarget. 2015 Aug 7;6(29):27519–36. doi: 10.18632/oncotarget.4823 (PMC4695006; doi:10.18632/oncotarget.4823)
Supplement: Supplementary file 1 [file oncotarget-06-27519-s001.pdf]

SUPPLEMENTARY DATA

1. Eagle H, Levine EM. Growth regulatory effects of cellular interaction. Nature. 1967; 213:1102–1106.
2. Abercrombie M. Contact inhibition and malignancy. Nature. 1979; 281:259–262.
3. Hamdane N, Stefanovsky VY, Tremblay MG, Nemeth A, Paquet E, Lessard F, Sanij E, Hannan R, Moss T. Conditional inactivation of Upstream Binding Factor reveals its epigenetic functions and the existence of a somatic nucleolar precursor body. PLoS Genet. 2014; 10:e1004505.
4. Yuan X, Zhou Y, Casanova E, Chai M, Kiss E, Grone HJ, Schutz G, Grummt I. Genetic Inactivation of the Transcription Factor TIF-IA Leads to Nucleolar Disruption, Cell Cycle Arrest, and p53-Mediated Apoptosis. Mol Cell. 2005; 19:77–87.

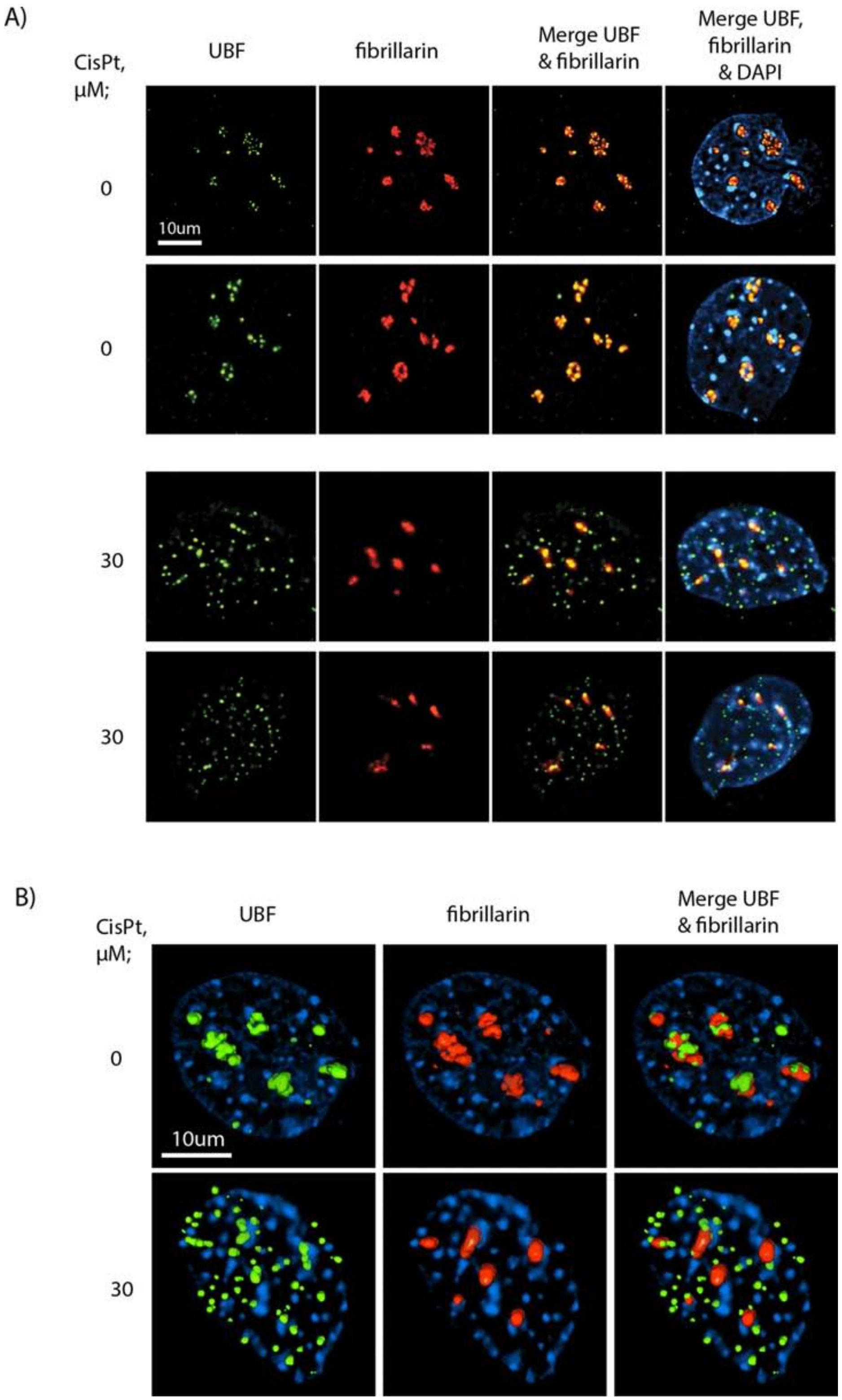

Supplementary Figure S1: Cisplatin treatment of MEFs induces displacement of UBF from the nucleolus. NIH3T3 cultures were treated with 30 µM cisplatin for 4 h in full medium or left untreated (0), then subjected to indirect immunofluorescence analysis of UBF (green), fibrillarin (red) and DNA stained with DAPI (blue). **A.** Single optical sections from image stacks of two untreated and two cisplatin treated cell nuclei are shown. **B.** 3D isosurface images generated from further examples of image stacks using Volocity (Perkin-Elmer), colours as in A).

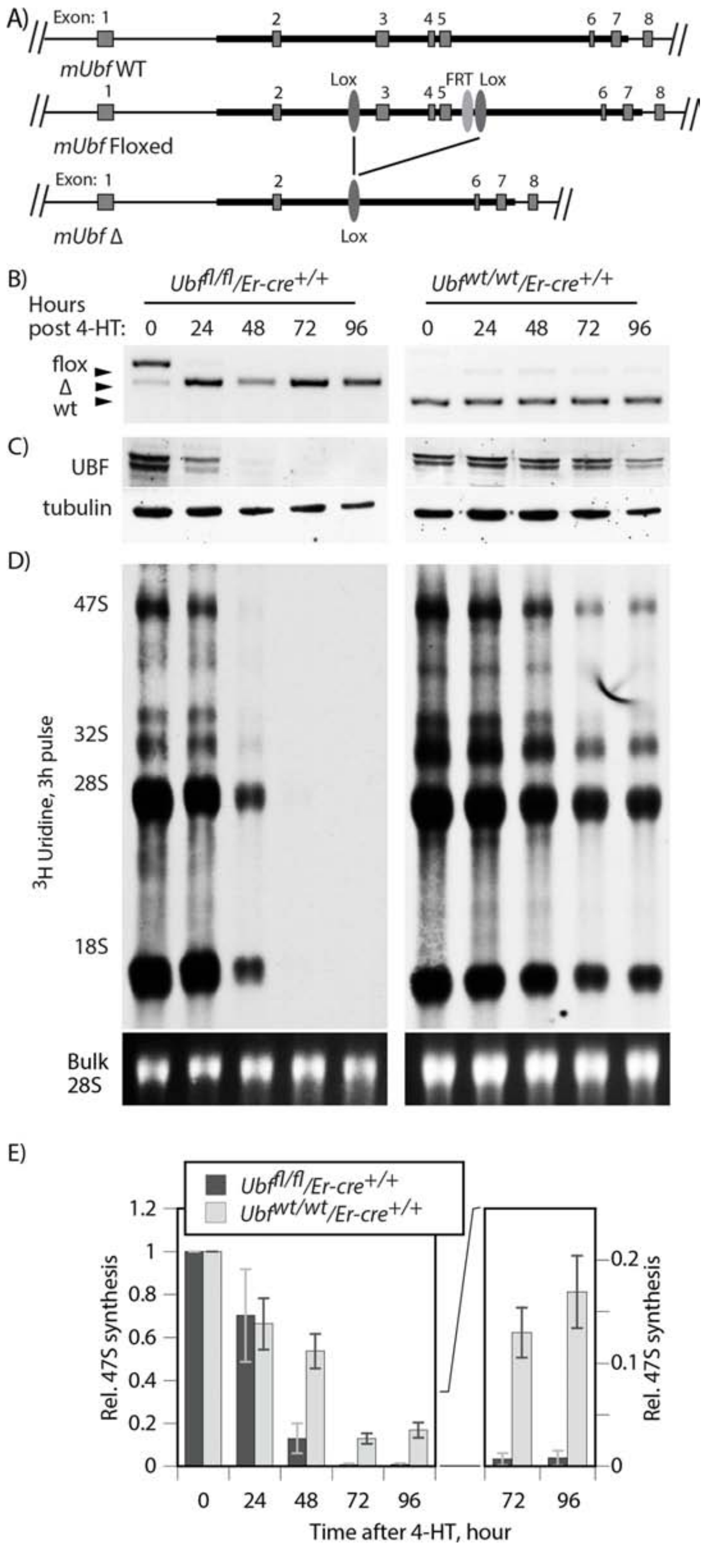

Supplementary Figure S2: Analysis of UBF loss in primary MEFs. A to C. Induction of Cre activity in *Ubf*$^{fl/fl}$/*Er-cre*$^{+/+}$ cells using only a 4 h treatment with 50 nM 4-hydroxytamoxifen (4-HT) induced the complete excision of the floxed *Ubf* exons by 24 h post 4-HT treatment (pHT), and the UBF protein was no longer detectable at 48 h pHT. **D and E.** Concomitantly, we observed a 90% reduction of rRNA synthesis, far in excess of that occurring as the control *Ubf*$^{wt/wt}$ /*Er-cre*$^{+/+}$ primary MEFs naturally reached confluence [1, 2], and rRNA synthesis was abolished by 72 h pHT.

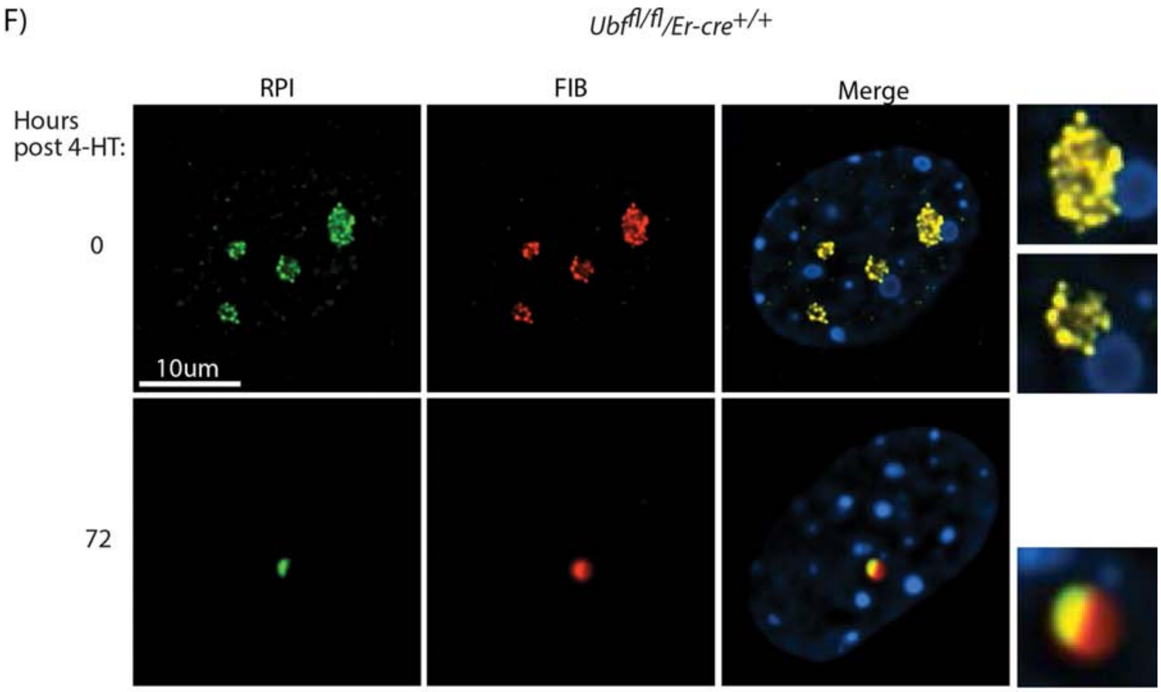

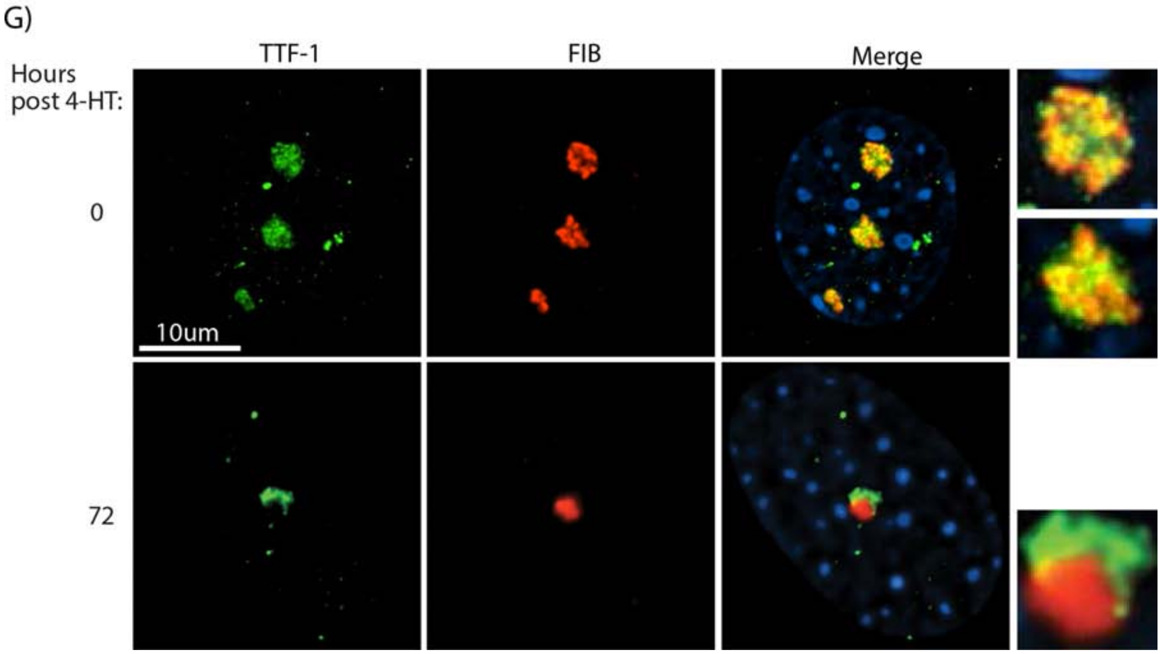

Supplementary Figure S2: (*Continued)* F and G. Further, loss of UBF caused the formation of a typical dense nucleolar protein body previously described in Ubf$^{fl/fl}$/Er-cre$^{+/+}$/SvT iMEFs [3]. Immunofluorescence analysis of F) RPI (large subunit, A194) and G) TTF-1 relative to Fibrillarin and DNA stained with DAPI before and after Ubf gene inactivation. The right-most panels show enlargements of single nucleolar bodies. Thus, UBF loss in primary MEFs recapitulated the effects previously observed in the SvT transformed MEFs (iMEFs).

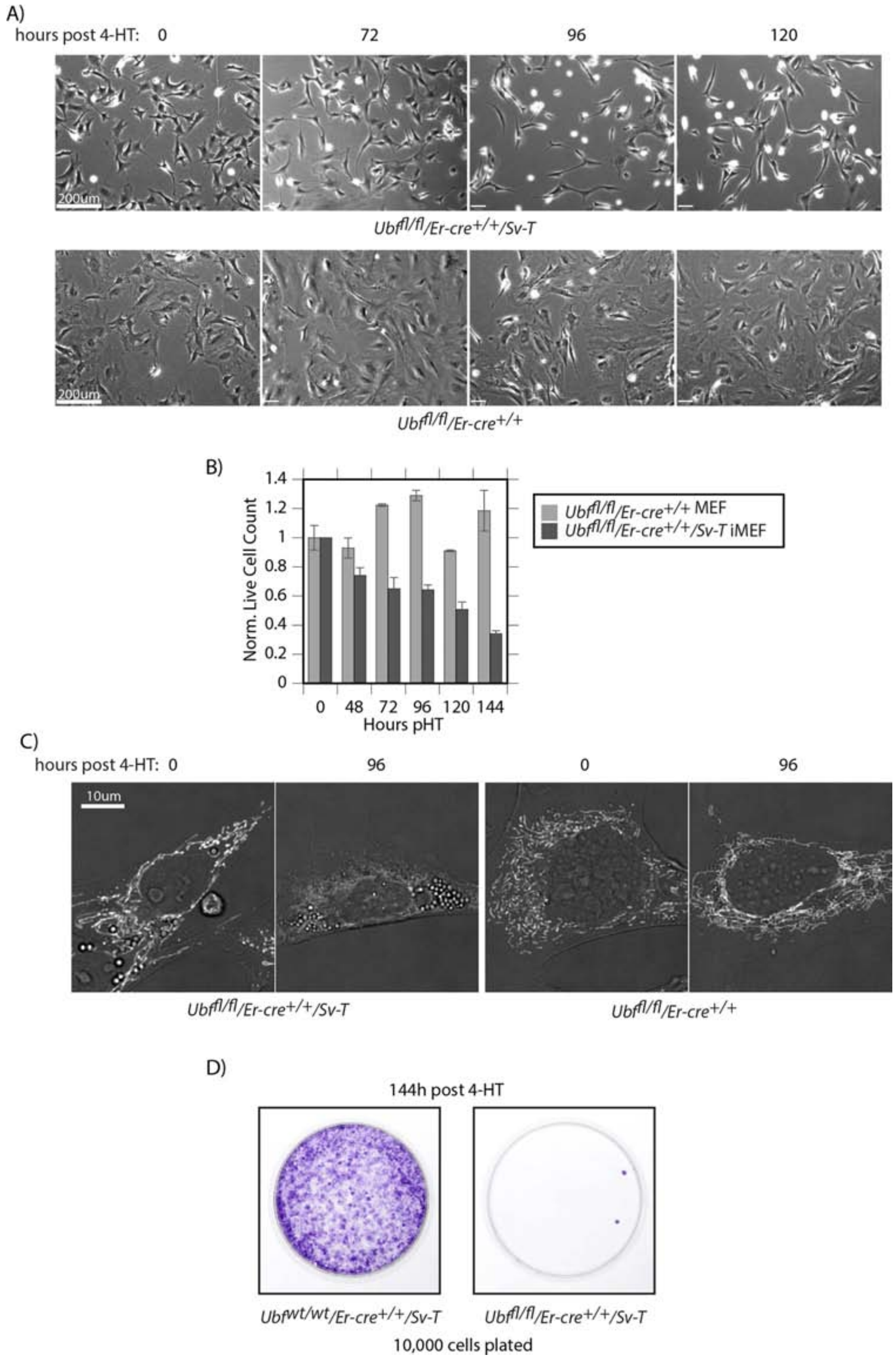

Supplementary Figure S3: Primary MEFs survive UBF loss while SV40Tt transformed iMEFs suffer cell death. A. UBF-loss causes morphological changes in *Ubf*$^{fl/fl}$/*Er-cre*$^{+/+}$*Sv-T* iMEFs but not in the *Ubf*$^{fl/fl}$/*Er-cre*$^{+/+}$ MEFs. B. Parallel analysis of iMEF and MEF viable cells during inactivation of the *Ubf* gene. The trypan blue negative cell count for each cell type has been normalized to that before activation of ER-Cre with tamoxifen (0 h pHT). C. MitoTracker staining of unfixed cultures of conditional iMEFs and MEFs before (0 h pHT) and after (96 h pHT) UBF loss. D. Colony Forming Assays for *Ubf*$^{fl/fl}$/*Er-cre*$^{+/+}$*Sv-T* and matched *Ubf*$^{wt/wt}$/*Er-cre*$^{+/+}$*Sv-T* iMEFs treated for 4 hours with 50 nM Tamoxifen at zero time and cultivated for a further 144 hours before fixing and staining cells.

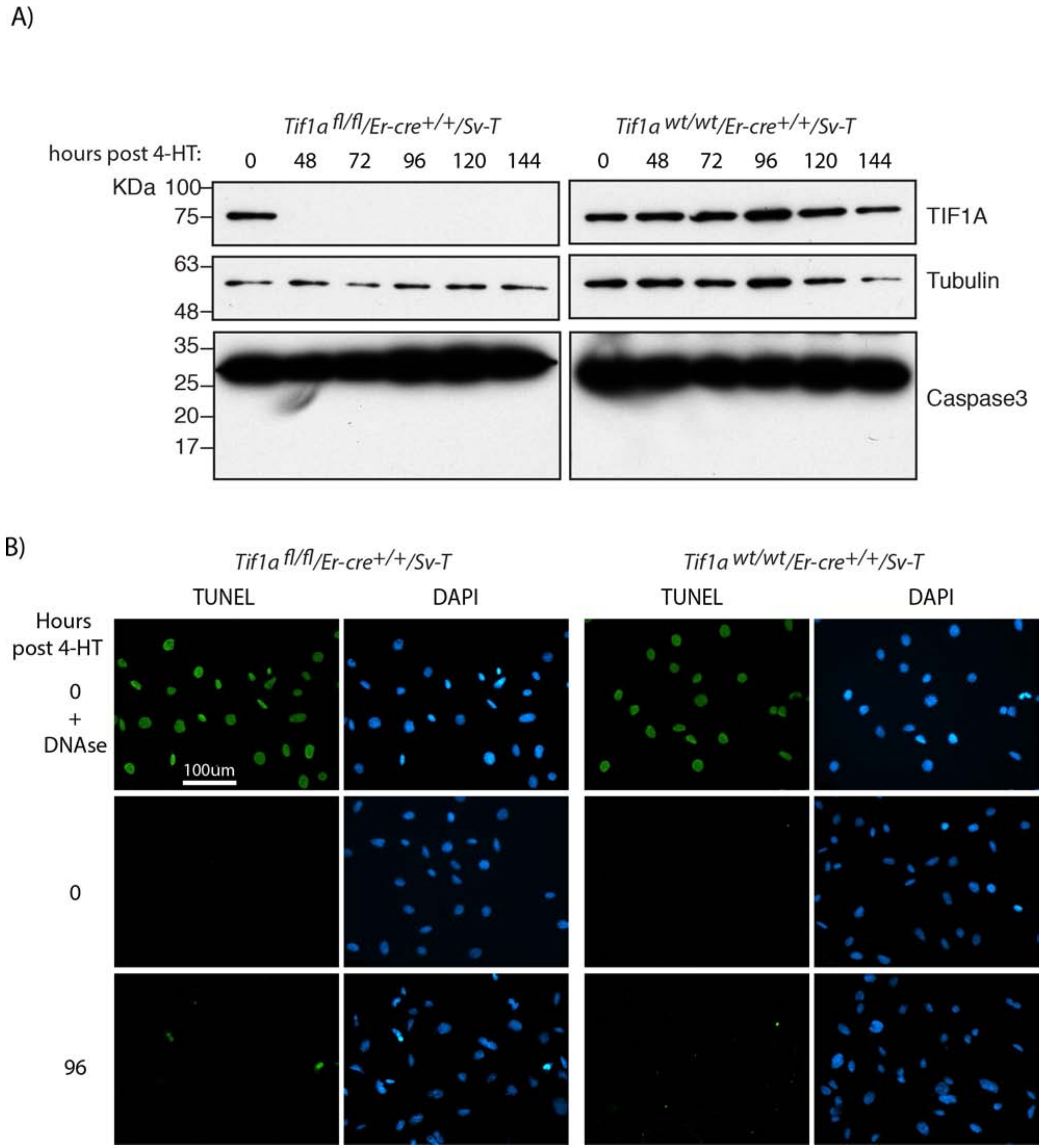

Supplementary Figure S4: TIF1A loss does not induce TUNEL positive apoptosis in SV40Tt transformed MEFs. **A.** Time course of 4-HT treatment of *Tif1a*$^{fl/fl}$/*Er-cre*$^{+/+}$/*Sv-T* and *Tif1a*$^{wt/wt}$/*Er-cre*$^{+/+}$/*Sv-T* MEFs derived from crosses of mice conditional for *Tif1a* [4]. The upper panels show the endogenous TIF1A levels and the central panel control Tubulin levels. The lower panel the status of Caspase 3 showing the p17 fragment is not detected. **B.** TUNEL assays of the same cells before and after TIF1A depletion. No TUNEL signal was detected unless cells were treated with DNase1 as control for the detection assay.

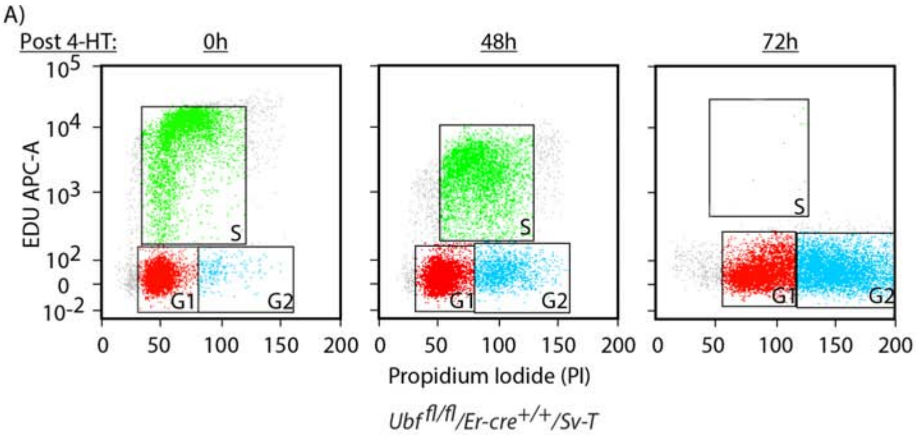

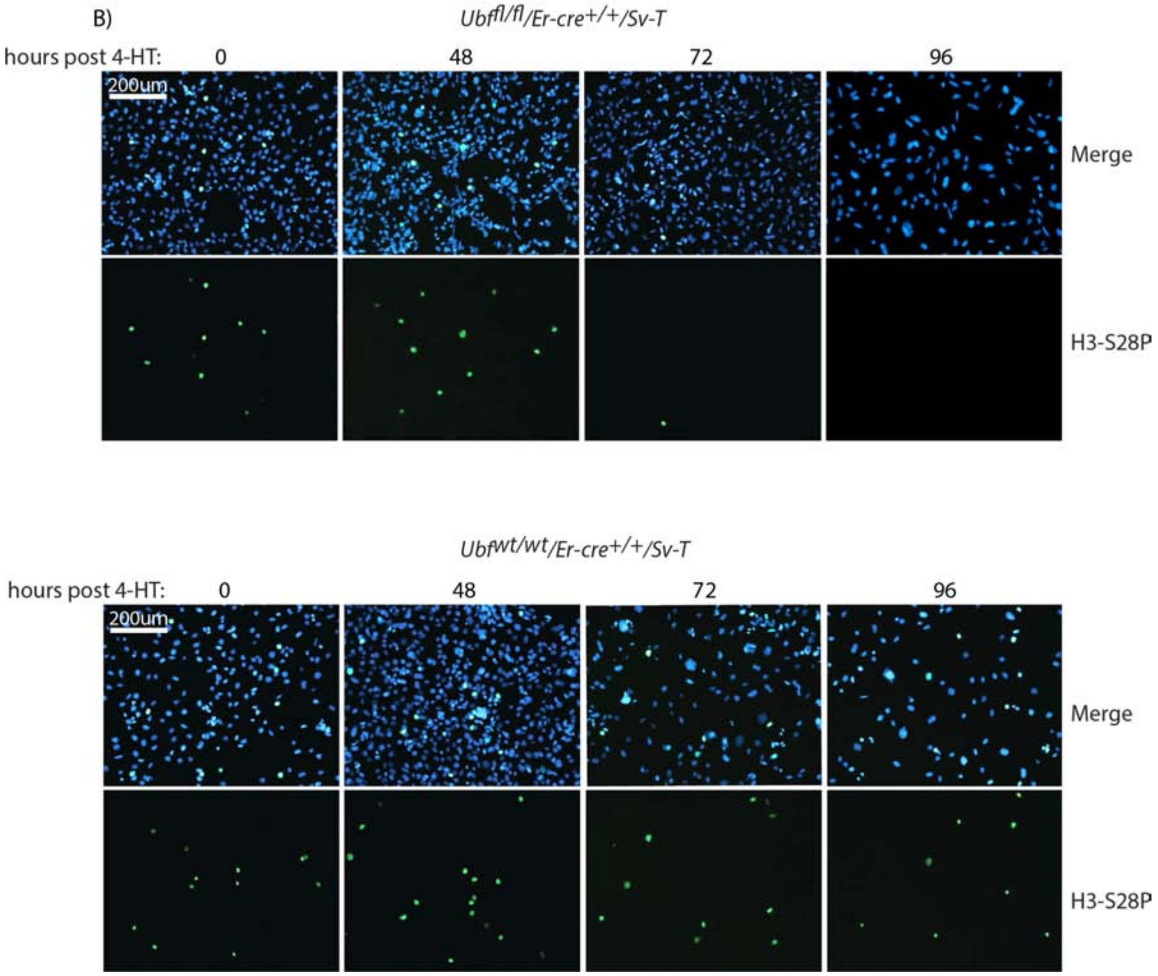

Supplementary Figure S5: UBF loss leads to a cell cycle arrest and to a loss of mitotic cells. A. *Ubf*$^{fl/fl}$*/Er-cre*$^{+/+}$*/Sv-T* iMEFs were analyzed for cell cycle distribution and **B.** mitotic index at the indicated times post 4-HT treatment. A) Shows an example of FACS analyses for active DNA replication (Click-iT® EdU) and DNA content (PI) at 0, 48 and 72 h post 4-HT treatment. B) Shows examples of immunofluorescence staining for phospho-serine 28 of histone H3. The mitotic index shown in Figure 5D was determined as the fraction of H3-S28 phospho-positive cells.

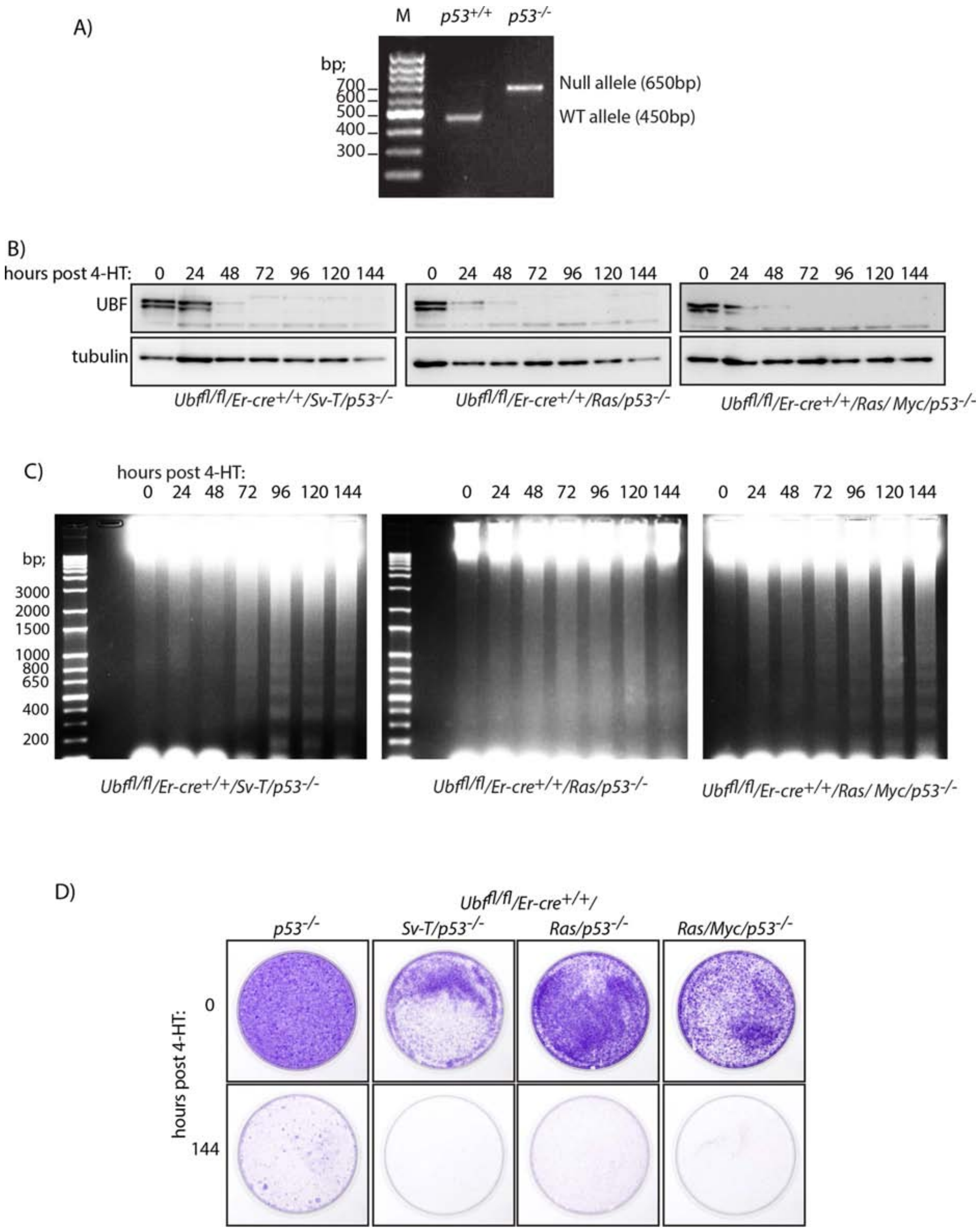

Supplementary Figure S6: p53-independent apoptosis is a general response to UBF loss in an oncogenic stress context. A. Genotyping of *p53*$^{-/-}$ and *p53*$^{+/+}$ iMEFs using the Jackson Laboratory protocol (http://jaxmice.jax.org/protocolsdb/f?p=116:2:0::NO:2:P2_MASTER_PROTOCOL_ID,P2_JRS_CODE:14323,002080) "M" indicates the DNA ladder size marker used. **B.** Time-course of UBF-loss in SV40Tt, Ras and Ras/Myc transformed p53-null UBF conditional MEFs treated with tamoxifen. **C.** Analysis of DNA degradation in the same cells and **D.** Colony forming capacity of these same iMEFs cultures before and 144 h after tamoxifen treatment.
